# RNA-Seq Analysis Reveals *Dendrobium officinale* Polysaccharides Inhibit Precancerous Lesions of Gastric Cancer through PER3 and AQP4

**DOI:** 10.1155/2021/3036504

**Published:** 2021-10-20

**Authors:** Yi Zhao, Hong-xia Huang, Su-yuan Tang, You-zhi Sun

**Affiliations:** ^1^Research Center for Differentiation and Development of Basic Theory of Chinese Medicine, Jiangxi University of Chinese Medicine, Nanchang, China; ^2^School of Traditional Chinese Medicine, Jiangxi University of Chinese Medicine, Nanchang, China

## Abstract

**Purpose:**

There has been mounting evidence that *Dendrobium officinale* polysaccharides (DOP), a traditional Chinese medicine, are a potential candidate treatment for N-methyl-N′-nitro-N-nitrosoguanidine- (MNNG-) induced precancerous lesions of gastric cancer (PLGC). However, the underlying mechanisms have not been adequately addressed.

**Method:**

We utilized RNA-Seq analysis to investigate possible molecular targets and then used Venn software to identify the differentially expressed genes (DEGs). Further, we analyzed these DEGs with core analysis, upstream analysis, and interaction network analysis by IPA software and validated the DEGs by real-time PCR and Western blot.

**Result:**

78 DEGs were identified from the normal control group (CON), the PLGC model group (MOD), and the DOP-treated group (DOP) by the Venn software. Further analysis of these DEGs, including core analysis, upstream analysis, and interaction network analysis, was performed by Ingenuity Pathway Analysis (IPA). The main canonical pathways involved were SPINK1 Pancreatic Cancer Pathway (−log (*P* value) = 4.45, ratio = 0.0667) and Circadian Rhythm Signaling (−log (*P* value) = 2.33, ratio = 0.0606). Circadian Rhythm Signaling was strongly upregulated in the model group versus the DOP group. CLOCK was predicted to be strongly activated (*z*-score = 2.236) in upstream analysis and induced the downstream PER3. In addition, the relative mRNA expression levels of seven DEGs (CD2AP, ECM1, AQP4, PER3, CMTM4, ESRRG, and KCNJ15) from RT-PCR agreed with RNA-Seq data from MOD versus CON and MOD versus DOP groups. The gene and protein expression levels of PER3 and AQP4 were significantly downregulated in the PLGC model and significantly increased by DOP treatment (9.6 g/kg).

**Conclusions:**

These findings not only showed DOP inhibits PLGC development by upregulating the PER3 and AQP4 gene and protein expression but also suggested that its mechanism of action involved modulating the Circadian Rhythm Signaling pathway.

## 1. Introduction

Gastric adenocarcinoma (GAC) is a common type of digestive tract cancer worldwide, which is the third cause of cancer-related deaths. It has an incidence rate and mortality rate of 10.6% (456,124 cases) and 13.6% (390,182 cases), respectively, in China in 2018 [1]. Precancerous lesions of gastric cancer (PLGC) are classified into intestinal metaplasia (IM) and dysplasia. The incidence rate of gastric cancer has been on a stable decline due to the eradication of *H. pylori*, improved standards of hygiene, and conscious nutrition [2]. Furthermore, reduced tobacco use and use of long-term proton pump inhibitors (PPIs), nonsteroidal anti-inflammatory drugs (NSAID) (statins and metformin), and rebamipide have been shown to play protective roles against GC, with reductions in the corresponding health care costs [3–5]. With the guidance of traditional Chinese medicine (TCM) theory, plant natural products were the main resources for cancer chemoprevention [6], and TCM can intervene at multiple targets and levels to treat PLGC [7].

*Dendrobium officinale* Kimura and Migo (*Dendrobium officinale*, called Tiepi Shihu in Chinese) belong to *Dendrobium* Sw, are famous traditional medicines, and are listed in the Chinese Pharmacopoeia. Recently, dried *D. officinale* leaves and flowers were used as food resources in Guizhou, Zhejiang, Yunnan, and Fujian provinces by their Health Commission [8]. According to TCM theories, *Dendrobium officinale* has the function of tonifying stomach and nourishing body fluid and has anticancer, antidiabetic, and immunomodulatory properties [9]. Chemical composition analysis reveals that *Dendrobium officinale* polysaccharides (DOP) are one of the main active compounds. Our research group first confirmed the prevention effect of DOP on precancerous lesions of gastric cancer (PLGC) and further found that DOP could regulate KEAP1/NRF-2 and Wnt/*β* catenin signaling pathway to prevent the development of cancer [10, 11].

RNA-Seq has been a preferred method of global gene expression analysis compared with microarrays given the large scale and complexity of transcriptomes [12]. It is widely used to explain the etiology of the disease or the therapeutic mechanism of drugs, especially in cancer; it will help to identify lineage-specific biomarker signatures for the cancer types [13], differentiate primary tumors, circulate tumor cells and metastases from a mouse xenograft model of cancer [14], and identify molecular pathways between cancer and normal tissue [13]. Since traditional Chinese medicine is a multitarget compound that affects multiple pathways, in this study, we performed RNA-sequencing (RNA-Seq) to more comprehensively explore which genes and pathways are related to the DOP treatment on the PLGC model. By targeting genes that have been significantly changed due to DOP, we were able to hopefully decipher some mechanisms of their action.

## 2. Materials and Methods

### 2.1. Reagents

Male Wistar rats (130 to 150 g) were provided by Beijing Vital River Laboratory Animal Technology Co., Ltd. (Certificate no. SCXK 2015-0002, Beijing, China). All rats were housed in the Animal Center of Jiangxi University of Chinese Medicine at 20 ± 2°C, humidity 40–60%, on a 12 : 12-hour light-dark cycle with free access to rodent feed and tap water. The animal study was reviewed and approved by the Animal Ethics Committee of Jiangxi University of Chinese Medicine (protocol code: JZLLSC2017016). *Dendrobium officinale* was purchased from Zhejiang Shou Xian Valley Medical Limited by Share Ltd. (Jinhua, Zhejiang, China). The detailed preparation, content, and molecular weight test by High-Performance Gel Permeation Chromatography (HPGPC) of DOP had been described previously [11]. MNNG was purchased from TCI (Shanghai) Development Co., Ltd. (Tokyo, Japan). Total RNA extraction kit was purchased from Promega Corporation (Promega, United States). Reverse Transcription PCR kit and RT-PCR amplification kit were purchased from Takara Biomedical Technology (Beijing, China); RIPA solution and BCA protein assay kit were purchased from Fisher Scientific (Santa Clara, United States). PVDF membranes were from Millipore, United States. Anti-PER3 antibody and anti-*β*-actin antibody were purchased from Abcam Company (Abcam, United States). Anti-AQP4 antibody was purchased from Proteintech Company (Proteintech, United States).

### 2.2. Animals and Experimental Design

After adaptive feeding for one week, seventy male rats were randomly divided into five groups by weight (*n* = 12 per group): normal control group (CON), the PLGC model group (MOD), and model treated with low dose (2.4 g/kg), middle dose (4.8 g/kg), and high dose (9.6 g/kg) of DOP. The administration dosage is according to China Pharmacopoeia 2015 Edition and the body surface area converted into rat dosage. The PLGC model group was given 150 *μ*g/ml MNNG in drinking water for 7 months and 0.1 ml of 10% NaCl once a week during the initial 20 weeks. During the pretrial period, rats were kept and fed in groups in the laboratory at room temperature (22–24°C) [15, 16]. DOP were administered to rats 2 weeks earlier. The body weights of rats were recorded every week throughout the 7 months. After seven months, all animals were sacrificed by intraperitoneal injection of thiopental after overnight fasting. The stomach was opened along the greater curvature on ice and kept in RNA preservation solution. In this experiment, we randomly choose stomach tissue from the normal control group (CON), the PLGC model group (MOD), and high dose (9.6 g/kg) of DOP (named DOP) (*n* = 3 per group) to program RNA-Seq and validated the result of RNA-sequencing by RT-PCR (*n* = 6 per group) and Western blot analysis (*n* = 3 per group).

### 2.3. RNA-Sequencing

Total RNA was extracted from gastric tissue samples. The purity and concentration of RNA were measured using a NanoDrop 2000 UV-Vis spectrophotometer (Thermo Scientific, USA). The Agilent Bioanalyzer 2100 system (Agilent Technologies, CA, USA) was utilized to assess the integrity of the RNA sample. Only RNA with RNA integrity values between 8 and 10 and purity between 1.8 and 2.1 were included in this study. RNA-sequence analysis was conducted by the BGISEQ-500 platform (BGI, Shenzhen, China). Three biological replicates were used for RNA-Seq.

### 2.4. RNA-Seq Data and Identification of DEGs

Clean reads were obtained by removing adapter-containing reads, poly-N-containing reads, and low-quality reads from the raw data and followed by mapping to the *Rattus norvegicus* genome sequence (Rnor 6.0) using HISAT2 (version 2.1.0) or STAR. The gene expression levels were estimated by fragments per kilobase of transcript per million fragments (FPKM). Gene expression levels were quantified using RNA-Seq by Expectation-Maximization (RSEM) [17]. Differentially expressed genes (DEGs) are defined with FDR ≤0.001 and fold change ≥1. Moreover, NOISeq software package analysis was used to screen differentially expressed genes between two groups. The cutoffs of probability ≥0.6 and |log2 ratio| ≥ 0.58 (ratio = 1.5) were applied to select DEGs.

### 2.5. Ingenuity Pathway Analysis (IPA) for Core Analysis, Upstream Analysis, and Interaction Network Analysis

Ingenuity Pathway Analysis, a web-based bioinformatics tool, was used for further pathway analysis. We uploaded the 78 genes excel file to IPA and then run the core analysis. Enrichment of the focus genes in networks in IPA was assessed via Fisher's exact test. Furthermore, the software identifies top functions associated with each network via enrichment scores (*z*-score), highlighting the predicted biological significance of the results [18, 19].

### 2.6. Validation of DEGs by Real-Time PCR

Based on the DEGs results, seven genes were selected for verification, and primers were designed using Primer Premier 5 software (Supplementary Table 1). The RNAs were reverse-transcribed to cDNA and then measured the relative mRNA expression by RT-PCR. The 2^−ΔΔCt^ method was used to perform the analysis while *β*-actin was utilized as the reference gene.

### 2.7. Validation of the Key Protein Expression by Western Blot

Three key proteins were selected for verification by western blot. The total protein samples from stomach tissues were extracted by RIPA solution and the protein content was determined using the BCA protein assay. 60 *μ*g of proteins was subjected to SDS-PAGE (15%) and then transferred to PVDF membranes. After blocking nonspecific binding sites with 5% dried milk for 1 hour at room temperature, the membranes were incubated overnight at 4°C with primary antibodies (1 : 1000). Immunoreactivity bands were quantified using the ChemiDoc™ XRS Imaging System (Bio-Rad Laboratories, USA). Intensity values expressed as the relative protein expression were normalized to *β*-actin.

### 2.8. Statistical Analysis

DEGs gene analysis was based on Ingenuity Pathway Analysis (IPA, http://www.ingenuity.com; Qiagen). RT-PCR and western blot data were presented as mean ± standard deviation (SD). Statistical analyses were performed using GraphPad Prism 8.0 software. A test of normality and homogeneity of variance was performed firstly, and data between two groups obeying normal distribution and homogeneous variance were evaluated by one-way analysis of variance (ANOVA). Otherwise, the nonparametric test was used. *P* ≤ 0.05 was considered statistically significant.

## 3. Results

### 3.1. Summary of Animal Experiment Results

The judgment of the model is based on HE and AB-PAS staining, and the result had been published [11]. The rats from the PLGC model showed severe atypical hyperplasia, intestinal metaplasia, and some nuclear fission, which indicated the model was built successfully. Following administration of DOP, especially high DOP, the mucosa was mild, and the degree of intestinal metaplasia was reduced. In the PLGC group, after AB staining, the middle and bottom of the gastric mucosa were positive, and the blue layer increased and thickened, indicating an acidic mucus; the upper was weakly PAS-positive with only a small amount of red staining. After treatment with DOP, the positive staining of AB at the bottom was reduced, and the blue layer was also reduced and thinned. The upper showed PAS staining was positive, and the red layer increased and thickened [10, 11].

### 3.2. Clean Reads Number and Mapped Reads

Sequencing data from the nine samples were mapped to the reference genome. As the summary of RNA-Seq analysis shows in Table 1, the clean reads number was about 24.0 million and the clean data rate was ≥99%. Additionally, as shown in Table 2, the results indicated total mapped reads were between 62% and 78%.

### 3.3. Detection of Genes and Transcripts

**Figure 1:**
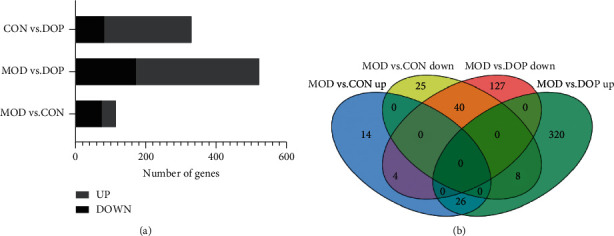
Statistical analysis of the gene expression detected by RNA-sequencing (RNA-Seq). (a) The number of differentially expressed genes (DEGs) and down- or upregulated DEGs. (b) Venn diagram of gene counts expressed in the MOD versus CON and MOD versus DOP groups.

**Figure 2:**
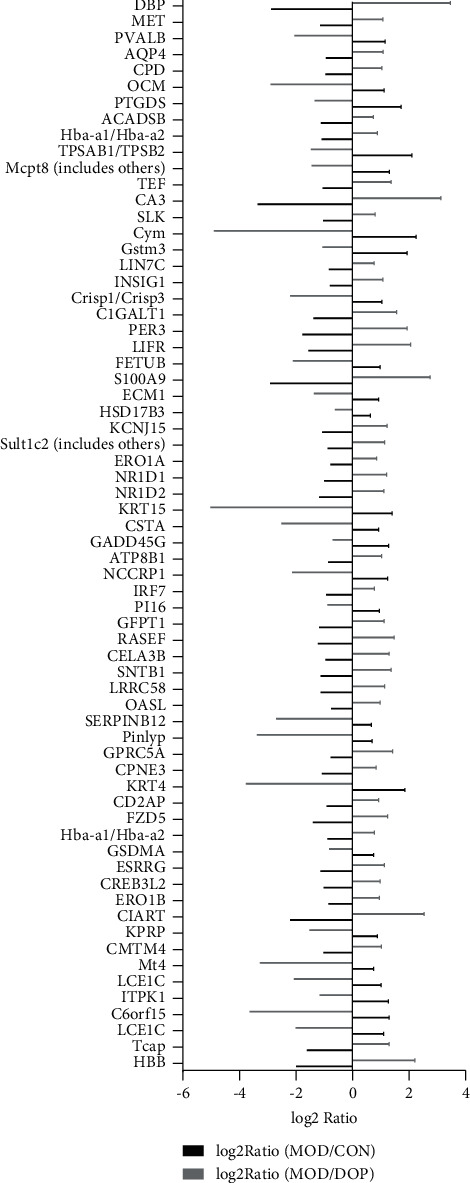
Significant common genes in MOD versus CON and MOD versus DOP groups. The ratio means the average expression of model/that of the control group and the same as model/DOP group.

Based on the RNA-Seq data, a total of 14517, 14516, and 14517 genes were identified in the MOD versus CON, MOD versus DOP, and CON versus DOP groups, respectively. After filtering these genes with the NOISeq method, the cutoffs of probability ≥0.6 and |log2 ratio| ≥ 0.58 (ratio = 1.5) were applied to select DEGs. The numbers of differentially expressed genes in MOD versus CON, MOD versus DOP, and CON versus DOP groups, respectively, were 117, 525, and 332 (Figure 1(a)). To understand the effects of DOP on the PLGC model, the MOD versus CON and MOD versus DOP groups were analyzed by the Venn software. 78 DEGs were commonly expressed in these two groups (Figure 1(b)), and there were 38 and 447 DEGs uniquely expressed in the control and DOP groups. For all the differentially expressed genes, 40 genes were significantly downregulated in the MOD versus CON and MOD versus DOP groups. On the other hand, 26 genes are significantly upregulated in the MOD versus CON group and MOD versus DOP group. A total of 66 genes were significantly altered by DOP treatment, as shown in Figure 2.

### 3.4. Ingenuity Pathway Analysis (IPA) for Core Analysis of 78 DEGs in MOD versus DOP Group

**Figure 3:**
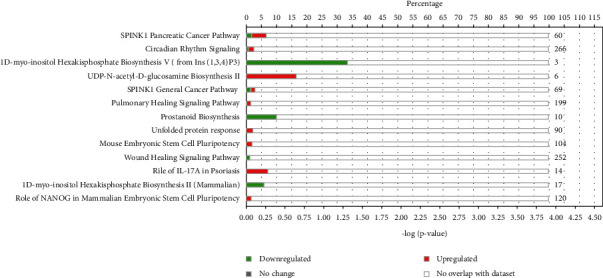
The main canonical pathways analysis by IPA in MOD versus DOP group. The red means upregulated, and the green means downregulated. The upper part of the *x*-axis means the percentage of genes that appeared during the experiment in the entire pathway.

To identify the gene networks and pathways affected by DOP in the PLGC model, the 78 differentially expressed genes in the MOD versus DOP group were uploaded into the IPA engine with their respective identifiers and log2FC values. The main canonical pathways were SPINK1 Pancreatic Cancer Pathway (−log (*P* value) = 4.45, ratio = 0.0667) and Circadian Rhythm Signaling (−log (*P* value) = 2.33, ratio = 0.0606). As shown in Figure 3, Circadian Rhythm Signaling was strongly upregulated in the MOD versus DOP group, which may suggests its involvement in the mechanism of DOP treatment to PLGC model.

### 3.5. Ingenuity Pathway Analysis (IPA) for Upstream Analysis in MOD versus DOP Group

**Figure 4:**
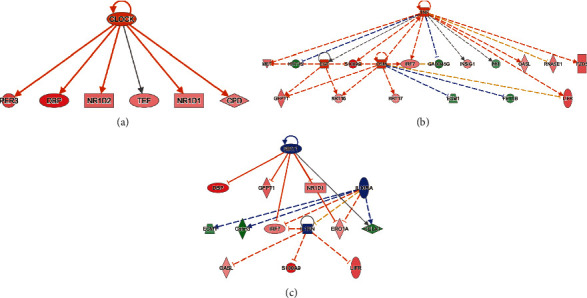
The predicted upstream regulators (activated and inhibited) and their target genes in MOD versus DOP group. (a) Predicted activated CLOCK and influence of the down target genes CPD, DBP, NR1D1, NR1D2, PER3, and TEF. DBP will be increased much more than the others. (b) Predicted activated OSM, TNF, and growth factor EGF.

To reduce the chance of false-positive significance due to random chance, IPA uses the activation *z*-score algorithm to predict activation or suppression of upstream modulators [20]. The results showed that 7 upstream factors were involved in regulating DEGs in the MOD versus DOP group. We then ranked the highest among the altered regulators and divided them with predicted activation state, that is, inhibited or activated. All these will hopefully help to reveal any causal connections between predicted upstream regulators and their target genes (Table 3). CLOCK was one of the genes in the Circadian Rhythm Signaling pathway, known as the transcription regulator, which was predicted to be strongly activated (*z*-score = 2.236). Its downstream target genes (PER3, DBP, NR1D2, TEF, NR1D1, and CPD) were also uniformly affected. Other upstream regulators such as cytokine OSM, TNF, and growth factor EGF were activated as well (*z*-score = 2.036, 2.064, and 2.166) (Figures 4(a) and 4(b)). SIRT1 was predicted to be strongly inhibited (*z*-score = −2.2), with 6 downstream target genes (CELA1, DBP, ERO1A, GFPT1, IRF7, and NR1D1) uniformly affected. Other upstream regulators such as IL1RN and IL10RA were also suppressed (*z*-score = −2.236 and −2) (Figure 4(c)).

### 3.6. The IPA Analysis of Gene-Gene Interactions

**Figure 5:**
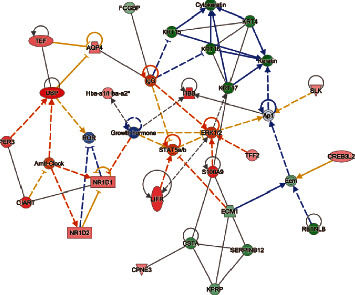
Gene-gene interaction analysis by IPA in MOD versus DOP group.

Interaction network analysis reveals interactions between molecules in datasets. IPA uses a network generation algorithm to divide the overall network of molecules into multiple subnetworks and then scores each network; the score is based on a hypergeometric distribution, and the negative logarithmic value of the significant level is obtained by Fisher's exact test. The analysis was composed of 7 networks, amongst which the top one is shown here. The network 1 diagram showed 25 focus molecules in data (Figure 5) (AQP4, CIART, CPNE3, CSTA, CREB3L2, DBP, ECM1, FCGBP, Hba-a1/Hba-a2, HBB, KPRP, KRT15, KRT16, KRT17, KRT4, LIFR, NR1D1, NR1D2, PER3, RETNLB, S100A9, SERPINB12, SLK, TEF, TFF2). The molecule activity predictor (MAP) predicted that DOP will upregulate PER3, DBP, NR1D2, TEF, NR1D1, CPD, and AQP4 based on mapped molecules.

### 3.7. Validation of DEGs by RT-PCR

**Figure 6:**
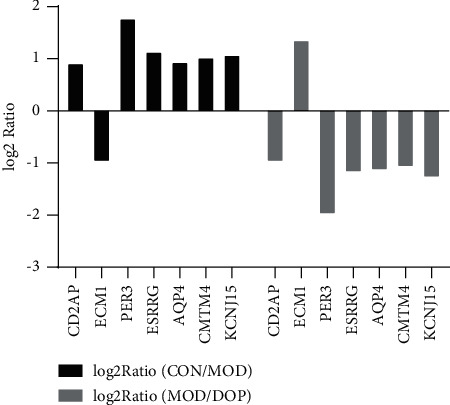
The log2 ratio of seven genes in RNA-Seq data.

**Figure 7:**
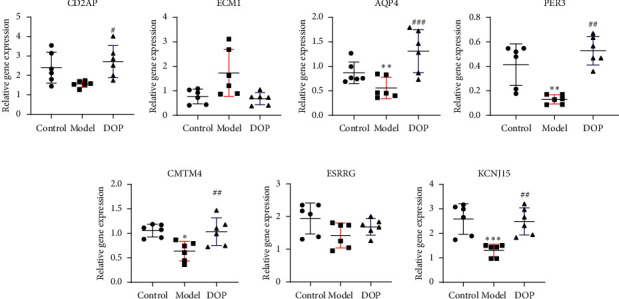
Relative mRNA expression of seven genes by RT-PCR (^*∗*^*P* < 0.05 and ^*∗∗*^*P* < 0.01 versus control group; ^##^*P* < 0.01 and ^###^*P* < 0.001 versus model group, *n* = 6).

To validate the key genes, we chose the seven genes because their biological functions are closely related to the occurrence and treatment of gastric cancer or precancerous lesions. The seven genes were CD2AP, ECM1, AQP4, PER3, CMTM4, ESRRG, and KCNJ15. Log2 ratios of CON/MOD and MOD/DOP are shown in Figure 6. The relative expression of mRNA was detected by real-time PCR. *β*-Actin was used as the reference gene. As shown in Figure 7, the relative expression changes in the real-time PCR data aligned with the RNA-Seq data, suggesting that the DEGs detected from our RNA-Seq analysis are valid.

### 3.8. Validation of DEGs by Western Blot

**Figure 8:**
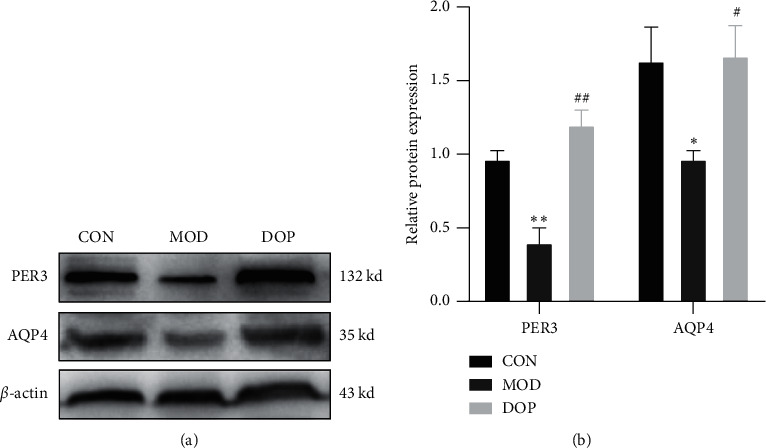
PER3 and AQP4 protein expression by western blot. (a) The protein expression by western blot. (b). The relative protein expression with *β*-actin (^*∗*^*P* < 0.05 and ^*∗∗*^*P* < 0.01 versus control group; ^#^*P* < 0.05 and ^##^*P* < 0.05 versus model group, *n* = 3).

To further investigate the molecular mechanism of DOP in the treated PLGC rat model, we measured the protein expression levels of PER3 and AQP4. PER3 is one of the primary components in Circadian Rhythm Signaling. As shown in Figure 8, the PER3 expression level was significantly decreased in the PLGC model as compared with the controls (*P* < 0.01). DOP (9.6 g/kg) strongly increased compared with the PLGC model group (*P* < 0.01). The level of AQP4 was decreased in the PLGC model group compared with the control (*P* < 0.05). DOP (9.6 g/kg) treatment significantly increased the expression levels of AQP4 (*P* < 0.05).

## 4. Discussion

Gastric dysplasia (GD) has been reported as a risk factor promoting the progression of GAC in patients. Up to 85% of high-grade (HGD) lesions eventually develop into GAC [2]. Traditional Chinese medicine could inhibit or postpone the progress of PLGC and therefore has been widely used in clinics. For example, Xiao Tan He Wei decoction reverses PLGC by inducing apoptosis via the NF-*κ*B pathway [21]. Wei Pi Xiao could attenuate early angiogenesis by inhibiting the expression of angiogenesis-mediating VEGF, a downstream target of the HIF-1*α* pathway [22, 23]. In addition, epigallocatechin-3-gallate (EGCG) and astragaloside IV had a preventive effect on the N-methyl-N′-nitro-N-nitrosoguanidine- (MNNG-) induced gastrointestinal cancer [24, 25]. Our research group has been studying the nourishing stomach yin medicine *Dendrobium officinale* in the treatment of PLGC. The previous studies demonstrated that *D. officinale* extraction (DOE) could prevent gastric carcinogenesis through regulating 8-OHdG, SOD, MDA, GSH-PX, and certain cytokines; upregulating Bax; and downregulating Bcl-2, EGF, EGFR, and S1P [26–28]. *Dendrobium officinale* polysaccharides (DOP) prevented PLGC through modulating KEAP1/NRF-2 and Wnt/*β* catenin signaling pathway [10, 11]. RNA-Seq has been a preferred method of global gene expression analysis compared with microarrays given the large scale and complexity of transcriptomes. To our knowledge, our present study is the first to report global transcription changes induced by DOP in the PLGC rat model.

In the present study, 525 genes in the gastric mucosa were observed between the MOD versus DOP groups, and 78 significantly changed genes were observed in MOD versus CON and MOD versus DOP groups. A core analysis through IPA software showed that Circadian Rhythm Signaling was upregulated. Furthermore, from the upstream analysis, CLOCK, which is known as the transcription regulator in Circadian Rhythm Signaling, was predicted to be strongly activated (*z*-score = 2.236), and its down target genes PER3, DBP, NR1D2, TEF, NR1D1, and CPD were uniformly affected. In our experiment, the mRNA and protein expression levels of PER3 were downregulated in the PLGC model and were upregulated in the DOP treatment group. Circadian rhythms were endogenously generated rhythms that occur with a periodicity of approximately 24 hours and play an important role in regulating the daily rhythms of human physiology and behaviors [29]. Studies have revealed that the circadian clock system consists of several genes including BMAL1, CLOCK (positive regulators), CRY1, CRY2, PER1, PER2, and PER3 (negative regulators) [30]. PER3 expression was downregulated in various cancers such as breast cancers, hepatocellular carcinomas, human lung cancer, and colorectal cancer [31–34]. PER3 overexpression will lead to the inhibition of cell proliferation and apoptosis [35]. Researchers reported that PER2 and CRY1 were significantly upregulated in gastric cancer. Moreover, the level of PER3 expression was negatively correlated with tumor stage and differentiation. Patients with high PER3 expression had better survival as compared to those with PER3-negative tumors [36]. Thus, we confirmed DOP could regulate PER3 via the Circadian Rhythm Signaling pathway. In addition, in gene-gene interactions analysis, the molecule activity predictor (MAP) showed DOP upregulated the expression of PER3, DBP, NR1D2, TEF, NR1D1, CPD, and AQP4 based on mapped molecules. Herein, we speculate that DOP could increase PER3 mRNA and protein expression to inhibit cell proliferation. These results pointed out PER3 may be the potential mechanism in DOP treatment through regulating the Circadian Rhythm Pathway. Therefore, further studies are necessary to fully address the functional role of PER3 in gastric cancer or precancerous lesions and whether such a role is exerted through the Circadian Rhythm Pathway.

The results also demonstrated that the mRNA and protein expression levels of AQP4 were downregulated in the PLGC model group, agreeing with prior research. After DOP treatment, an upregulation of AQP4 was observed from both western blot analysis and network 1, suggesting DOP is able to mitigate malignant genotypes. However, further investigation is needed to make clear how DOP treatment could reverse the repressed AQP4 expression in the PLGC model. Aquaporins (AQPs) are a family of integral membrane proteins that are expressed in all living organisms and play vital roles in transcellular and transepithelial water movement. They have been reported to also play a crucial role in cell migration, proliferation, and angiogenesis in different cancer cells [37]. Aquaporin 4, located primarily in gastric parietal cells and colonic surface epithelium, has been shown to have possible roles in gastric acid and enzyme secretions [38, 39]. AQP4 was absent in human carcinoma tissue in contrast with healthy tissue, indicating its downregulation during gastric tumorigenesis [40]. Indeed, AQP4 protein and mRNA expression levels in gastric cancer tissue were significantly lowered than those in normal gastric tissue [41, 42]. Therefore, active expression of AQP4 will restrain cell migration and proliferation, which was another way to explain the mechanism of DOP in inhibiting MNNG-induced PLGC in the rat model.

## 5. Conclusion

RNA-Seq revealed the mechanism of DOP to inhibit MNNG-induced PLGC in the rat model; the main canonical pathways were SPINK1 Pancreatic Cancer Pathway and Circadian Rhythm Signaling. DOP significantly influenced 66 gene expressions; the most obvious effect is to increase the protein and gene expression of PER3 and AQP4 in the gastric precancerous lesion model. This study implied that modulating the Circadian Rhythm Signaling pathway might be a potential mechanism of DOP in treating PLGC.

## Figures and Tables

**Table 1:** A summary of the RNA-Seq analysis.

Sample	Raw data size (bp)	Raw reads number	Clean data size (bp)	Clean reads number	Clean data rate (%)
Control 1	1200338700	24006774	1198478650	23969573	99.84
Control 2	1200816650	24016333	1199033750	23980675	99.85
Control 3	1200810750	24016215	1199003450	23980069	99.84
Model 1	1206815500	24136310	1196690550	23933811	99.16
Model 2	1206818250	24136365	1203062050	24061241	99.68
Model 3	1200644450	24012889	1189440500	23788810	99.06
DOP1	1200679900	24013598	1198722550	23974451	99.83
DOP2	1200849900	24016998	1198667200	23973344	99.81
DOP3	1200291450	24005829	1198091850	23961837	99.81

**Table 2:** Total mapped reads.

Sample	Total reads	Total mapped reads (%)	Unique match (%)	Multiposition match (%)	Total unmapped reads (%)
Control 1	23969573	66.62	65.33	1.29	33.38
Control 2	23980675	75.03	74.16	0.87	24.97
Control 3	23980069	63.21	61.85	1.36	36.79
Model 1	23933811	74.33	73.11	1.23	25.67
Model 2	24061241	78.19	77.33	0.87	21.81
Model 3	23788810	66.92	65.5	1.42	33.08
DOP1	23974451	71.76	70.76	1.01	28.24
DOP2	23973344	67.37	66.12	1.25	32.63
DOP3	23961837	63.88	62.38	1.5	36.12

**Table 3:** The upstream regulators' analysis by IPA in MOD versus DOP group.

Upstream regulator	Predicted activation state	Predicted activation state	Activation *z*-score	*P* value of overlap	Target molecules in dataset
OSM	Cytokine	Activated	2.036	0.0000824	ECM1, FETUB, GADD45G, GFPT1, IRF7, KRT16, KRT17, LIFR, S100A9
TNF	Cytokine	Activated	2.064	0.0403	FZD5, GADD45G, INSIG1, IRF7, KRT15, LIFR, MET, Mt1, OASL, RNASE1, S100A9
EGF	Growth factor	Activated	2.166	0.0313	GFPT1, KRT15, KRT16, MET, S100A9
CLOCK	Transcription regulator	Activated	2.236	0.00019	CPD, DBP, NR1D1, NR1D2, PER3, TEF
IL10RA	Transmembrane receptor	Inhibited	−2.236	0.00337	CELA1, ECM1, ERO1A, Gstm3, IRF7
SIRT1	Transcription regulator	Inhibited	−2.2	0.00267	CELA1, DBP, ERO1A, GFPT1, IRF7, NR1D1
IL1RN	Cytokine	Inhibited	−2	0.000856	IRF7, LIFR, OASL, S100A9

## Data Availability

All the relevant data are presented within the manuscript.

## Supplementary Materials

Supplementary Materials Table 1: RT-PCR primer information.
