# Supplementary material for: RNA-Seq Analysis Reveals Dendrobium officinale Polysaccharides Inhibit Precancerous Lesions of Gastric Cancer through PER3 and AQP4
Source: Evid Based Complement Alternat Med. 2021 Oct 20;2021:3036504. doi: 10.1155/2021/3036504 (PMC8550840; doi:10.1155/2021/3036504)
Supplement: Supplementary Materials — Table 1: RT-PCR primer information. [file 3036504.f1.docx]

Table 1. RT-PCR primer information.

| Genes | Sense (5’-3’) | Antisense (5’-3’) | Size (bp) |
| --- | --- | --- | --- |
| CD2AP | TGGTAGCCAGGAACTCAAAAG | TGGAGAATGTCCACCATTGA | 143 |
| Ecm1 | CCGTGACCAGTTCTTACCCC | CTGAAACCTTGAAGGCTCCCT | 108 |
| AQP4 | TCA GCA TCC AAG AAA CCA TA | ACC TCC CAC CAG ACT CC | 147 |
| PER3 | CTCACACCGCTCCAACTCAACTC | ATGTCTCTTCTGCTCCCTGTCTCC | 131 |
| CMTM4 | AAGGTCGCCCAAGTGATTTTAGCC | AAGACCCCAGTCACCACAAATGC | 131 |
| ESRRG | CCCGGCCACGAATGAATGTG | GACCTCCACGCACTCGTCA | 128 |
| KCNJ15 | CGCATTCTCCTCAACCAGGCTAC | GGGCTCGTCTCATCCAACACATG | 110 |
| TFF2 | CCAAACCAAGCGTCGGAACAATG | TGTCGGCTGGCACAGTCCTC | 95 |
